# Morphological Variations of the Extracranial Internal Carotid Artery: Prevalence, Age and Sex-Related Associations, and Imaging Insights From Magnetic Resonance Angiography

**DOI:** 10.7759/cureus.102300

**Published:** 2026-01-26

**Authors:** Ashish Kumar, Priyanka Sharma, Islam Khan, Manoj M Kulkarni, Hetal Vaishnani, Ashutosh Patel, Parth Chandubhai Savaliya

**Affiliations:** 1 Anatomy, Smt. B.K. Shah Medical Institute & Research Centre, Vadodara, IND; 2 Physiology, Govt. Medical College, Alwar, IND; 3 Radiology, Smt. B.K. Shah Medical Institute & Research Centre, Vadodara, IND

**Keywords:** coiling, dolichoarteriopathy, internal carotid artery (ica), kinking, magnetic resonance angiography (mra), tortuosity

## Abstract

Introduction

Dolichoarteriopathies of the extracranial internal carotid artery (ICA), such as tortuosity, kinking, and coiling, are frequently encountered vascular variations that may influence cerebral hemodynamics and clinical outcomes.

Purpose

This study aims to determine the prevalence and types of extracranial internal carotid artery variations and assess their relationship with age and sex using non-invasive magnetic resonance angiography (MRA).

Methods

This cross-sectional observational study was conducted at a tertiary care centre from June 2024 to July 2025 and included 100 consecutive patients (18-90 years, both sexes) referred for head and neck magnetic resonance angiography. Patients with prior cerebrovascular disease, central nervous system neoplasms, recent major head trauma, or suboptimal imaging were excluded. Three-dimensional time-of-flight MRA was performed, and extracranial ICAs were independently evaluated by two radiologists for dolichoarteriopathy type and severity. Statistical analysis included frequency distribution and Chi-squared testing to assess associations with age and sex.

Results

A total of 197 extracranial ICAs were analyzed, of which 70.05% demonstrated dolichoarteriopathy. Tortuosity was the most common variant (69.56%), followed by kinking (23.18%) and coiling (7.24%). The prevalence was significantly higher in patients older than 40 years compared to those aged 40 years or younger (89.25% vs. 39.47%, p=0.001). Although dolichoarteriopathies were more frequent in females than males (74.62% vs. 67.69%), the difference was not statistically significant. Morphological patterns varied significantly with age but showed no significant association with sex or laterality.

Conclusion

ICA dolichoarteriopathies are common, predominantly age-related anomalies with potential clinical relevance. Non-invasive MRA provides a reliable means for their detection and characterization. Systematic assessment of ICA morphology is recommended, especially in older populations or individuals with vascular comorbidities. Prospective studies integrating clinical outcomes and hemodynamic analysis are warranted to further define their prognostic significance

## Introduction

The internal carotid artery (ICA) originates from the common carotid artery at the level of the upper border of the thyroid cartilage and ascends through the parapharyngeal space to enter the skull via the carotid canal of the temporal bone [1]. Normally, the extracranial segment of ICA (EICA) follows a relatively straight course and does not give rise to collateral branches in the neck [2]. However, variations in the course and geometry of the EICA are frequently encountered and have been widely documented in anatomical, radiological, and clinical studies [3,4].

Radiographic classification of EICA morphological variations was first systematically described by Weibel and Fields, who categorized these anomalies into tortuosity, kinking, and coiling based on arterial geometry [5]. Tortuosity refers to gentle C- or S-shaped elongations of the artery, kinking is characterized by an acute angular bend of varying severity, and coiling describes exaggerated elongation with looped or circular configurations [5-7]. Collectively, these elongation-related anomalies are referred to as dolichoarteriopathies of the EICA [6,8].

Dolichoarteriopathies are relatively common, with reported prevalence ranging from approximately 30% in population-based studies to nearly 58% in hospital-based cohorts [9,10]. This wide variability is attributed to differences in imaging techniques, diagnostic criteria, and demographic characteristics of study populations. Age-related vascular remodeling, degeneration of elastin fibers, and chronic hypertension have been identified as important contributors to the development and progression of these anomalies, which frequently occur bilaterally in older individuals [11-15].

Previous studies have reported inconsistent findings regarding sex-related differences in the prevalence and morphology of EICA dolichoarteriopathies, highlighting the need for further evaluation [9,10].

Although most EICA morphological variations are asymptomatic and detected incidentally, marked tortuosity, kinking, or coiling may alter cerebral hemodynamics and contribute to symptoms such as dizziness, syncope, transient ischemic attacks, ischemic stroke, and other manifestations of cerebrovascular insufficiency [10,11].

These variations are also of particular clinical importance during head and neck surgeries and endovascular procedures, as aberrant arterial courses may increase the risk of iatrogenic injury [1,4,16].

Evaluation of the EICA can be performed using several imaging modalities, including contrast-enhanced computed tomography, CT angiography, and magnetic resonance angiography (MRA). Among these, MRA offers high-resolution three-dimensional visualization of vascular anatomy without ionizing radiation, making it a reliable and non-invasive technique for assessing EICA morphology and geometric variations [16].

The present study evaluates the morphological variations of the EICA using magnetic resonance angiography, determines their prevalence, and analyzes their association with age and sex.

## Materials and methods

Study population

This cross-sectional observational study was conducted in the Department of Radiology at Dhiraj Hospital, Smt. B.K. Shah Medical Institute and Research Center, Vadodara, Gujarat, India, with recruitment between June 2024 and July 2025. Consecutive patients referred for MRA of the head and neck were assessed for inclusion. Eligible participants were aged between 18 and 90 years, of either sex, and underwent MRA. Patients were excluded if they had a documented history of cerebrovascular disease (such as stroke, intracranial aneurysm, or arteriovenous malformation), clinical or radiological evidence of central nervous system neoplasm, history of significant head trauma (within the past year or resulting in lasting neurological deficit), or if the MRA images obtained were of insufficient quality owing to motion or flow artifacts. The study cohort consisted of 100 patients who met all eligibility requirements.

Study design

All participants underwent three-dimensional time-of-flight (3D TOF) MRA of the head and neck using a 1.5 Tesla Philips MRI scanner (Philips, Amsterdam, Netherlands) equipped with a dedicated head and neck coil. Imaging parameters included a repetition time/echo time (TR/TE) of approximately 20/3.4 ms, slice thickness of 1 mm, field of view of 200-220 mm, and flip angle of 15-20°. Post-processing involved maximum intensity projection (MIP) and curved planar reformation (CPR) reconstructions to enhance visualization of the EICA [17-19].

**Figure 1:**
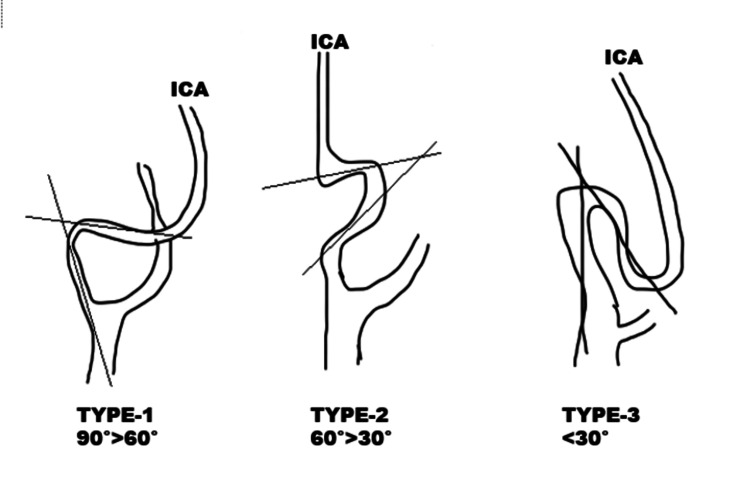
Metz classification of severity of kinking (mild, moderate, severe) Two lines drawn along the axis of the internal carotid artery show an angle of curvature of the vessel less than 90° (type 1), 60° (type 2), or 30° (type 3) ICA - internal carotid artery Figure created by the authors.

**Figure 2:**
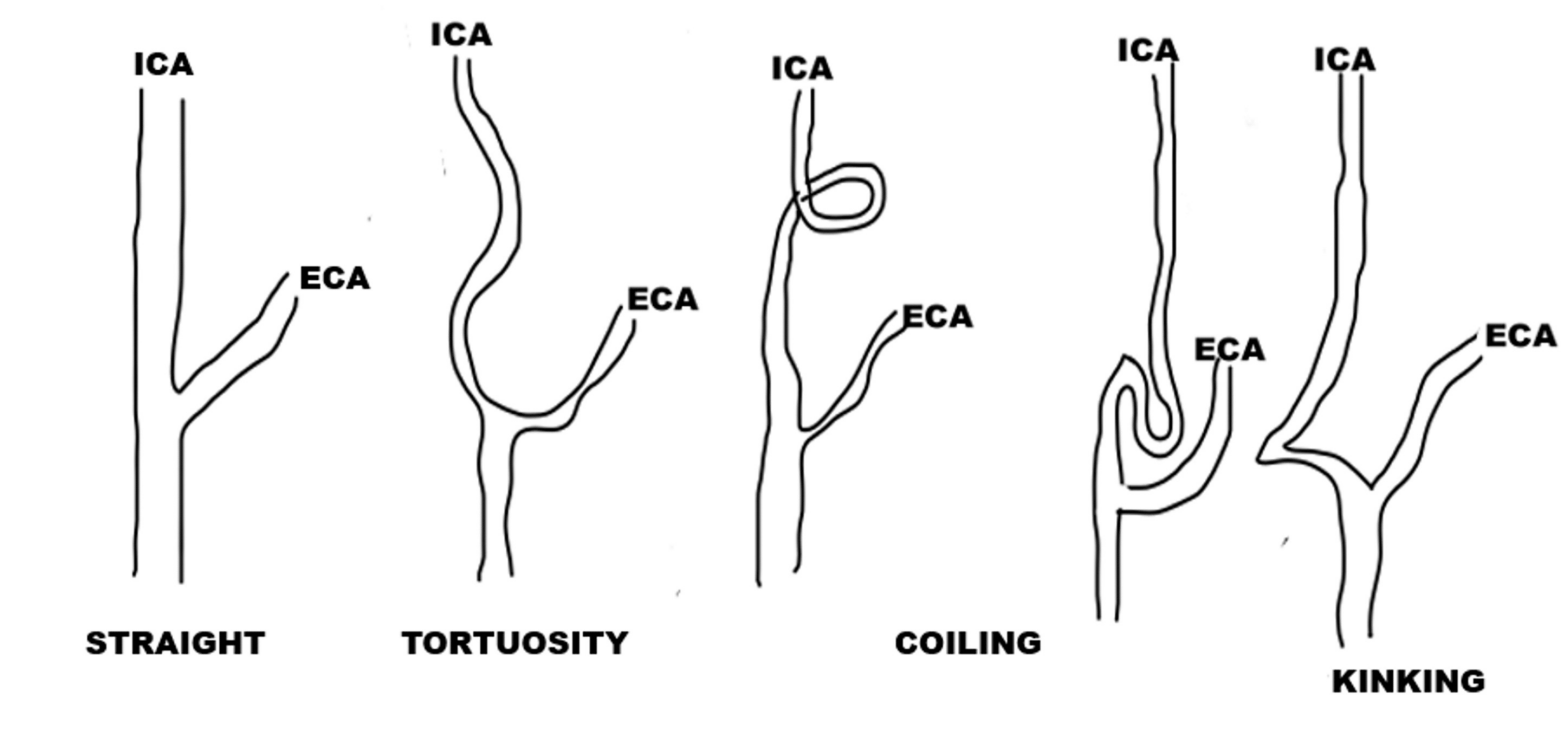
Schematic drawing of morphological classification of carotid artery (lateral view) ICA - internal carotid artery; ECA - external carotid artery Figure created by the authors.

The study examined the demographic characteristics of the patients, including age, sex, and medical history. To evaluate the variations in the EICA and its anatomical relationships in MRA, specific measurements and criteria were utilized. Variations such as coiling were defined as an artery path that completes a full 360° rotation. Kinking was characterized as a morphological anomaly of the artery with sharp angulation (not exceeding 90°). Kinking curves were categorized according to the Metz classification based on the angle's severity [20] (Figure 1): Metz 1 or mild kinking (<90°), Metz 2 or moderate kinking (<60°), and Metz 3 or severe kinking (< 30°). Straight type and tortuosity means S- or C-shaped elongation of the vessel without sharp angulation were assessed qualitatively through visual inspection of the reconstructed images (Figure 2) [19].

Image interpretation was performed independently by two radiologists, each with more than five years of experience in vascular imaging, who were blinded to the clinical details and demographic data of the participants. In cases of disagreement, discrepancies were resolved by consensus review. Demographic and clinical data were extracted from hospital electronic medical records. Both sides of the EICA were assessed from the carotid bifurcation to the skull base.

The Institutional Research Ethics Committee of Sumandeep Vidyapeeth deemed to be University granted approval for the study protocol, and the radiology department secured written informed consent from each participant.

Statistical analysis

Data were analyzed using SPSS version 25 software (IBM Inc., Armonk, NY, USA). Continuous variables are presented as mean ± SD, and categorical variables as frequencies and percentages. The distribution of continuous variables was examined to ensure that the data were approximately symmetrical and free from major outliers. Variability across comparison groups was also assessed. Statistical analyses were chosen accordingly: parametric methods were used when data met these assumptions, while non-parametric methods were applied when assumptions were not satisfied. Associations between EICA morphological variations and age or sex were assessed using Chi-square or Fisher's exact test, with odds ratios (OR) and 95% confidence intervals (CI). Age differences were tested using a t-test or the Mann-Whitney U test. Kinking severity (Metz classification) was analyzed across age and sex. A p-value <0.05 was considered significant.

## Results

Demographic characteristics

A total of 100 patients were included in the study. The mean age of the study population was 46.93 ± 16.17 years, with an age range of 18 to 90 years. Among them, 38 patients (38%) were aged ≤40 years, while 62 patients (62%) were older than 40 years. The gender distribution showed a male predominance, with 66 males (66%) and 34 females (34%) (Table 1).

**Table 1:** Presents the demographic characteristics of the study population This table summarizes the demographic profile of the study participants (n=100), including age distribution and gender. Age is expressed as mean ± standard deviation (SD) and categorized into ≤40 years and >40 years. Gender distribution is presented as number and percentage (%)

Variable	Category	n (%)
Age (years)	Mean ± SD	46.93 ± 16.17
	Range	18-90
	≤ 40 years	38 (38%)
	> 40 years	62 (62%)
Gender	Male	66 (66%)
	Female	34 (34%)

Prevalence of morphological variations of the EICA

**Figure 3:**
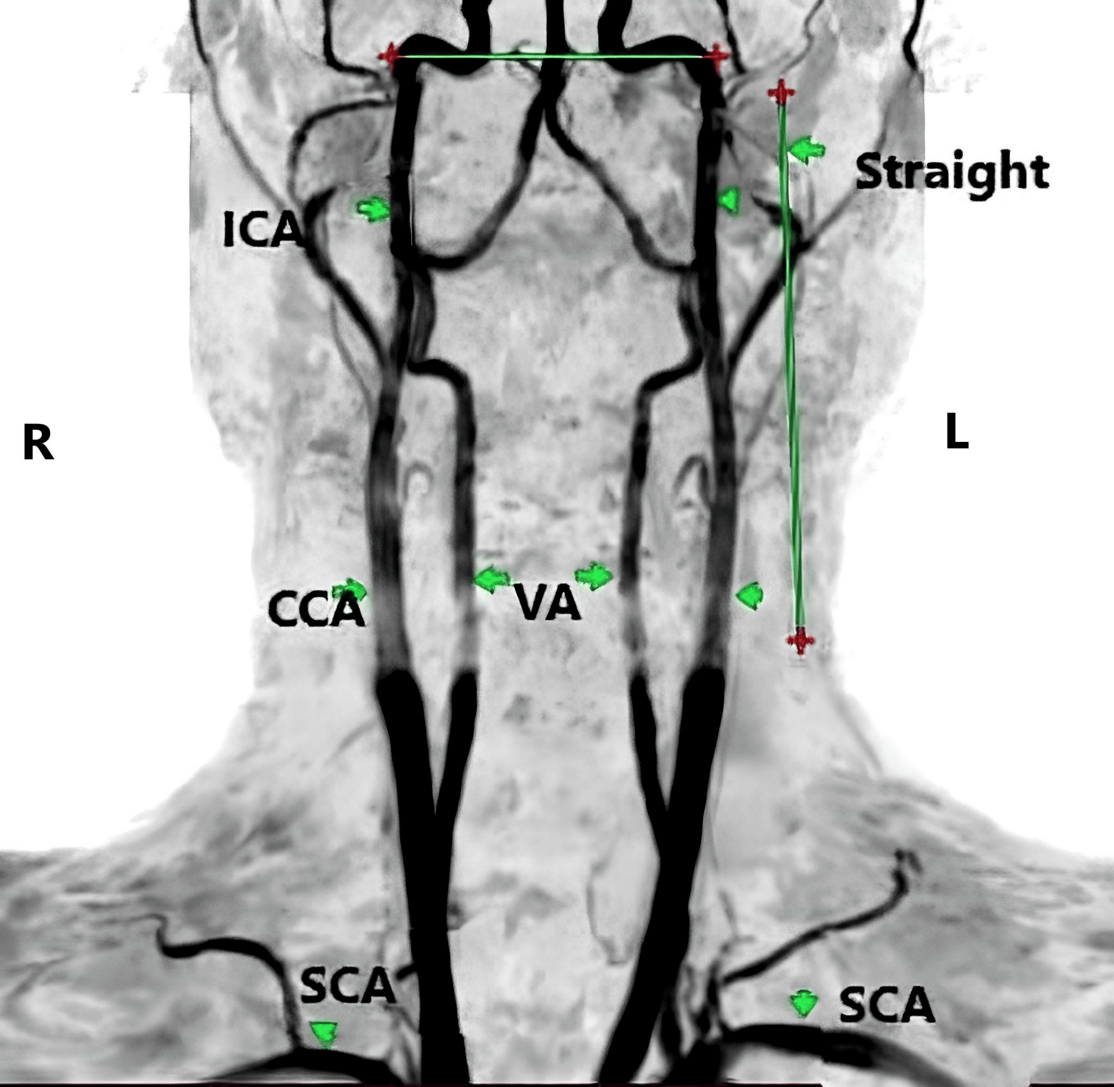
Bilateral straight type with vertical course MRA of the neck vessels demonstrating dolichoarteriopathy of EICA. EICA - extracranial segment of the internal carotid artery; SCA - subclavian artery; CCA - common carotid artery; VA - vertebral artery

**Figure 4:**
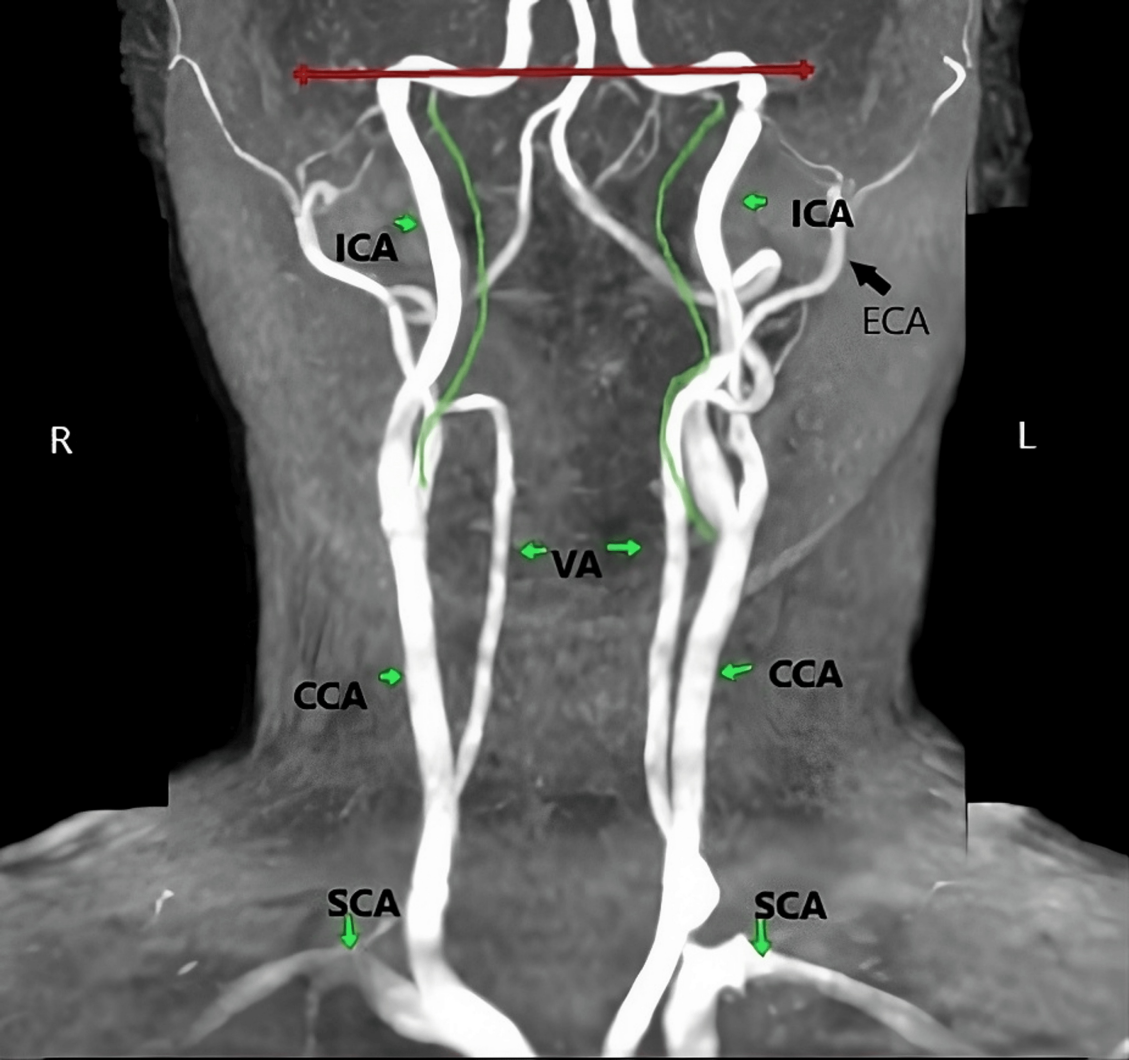
Bilateral tortuous type with smooth S-shaped elongation MRA of the neck vessels demonstrating dolichoarteriopathy of EICA. EICA - extracranial segment of the internal carotid artery; SCA - subclavian artery; CCA - common carotid artery; ECA - extracranial artery; VA - vertebral artery

**Figure 5:**
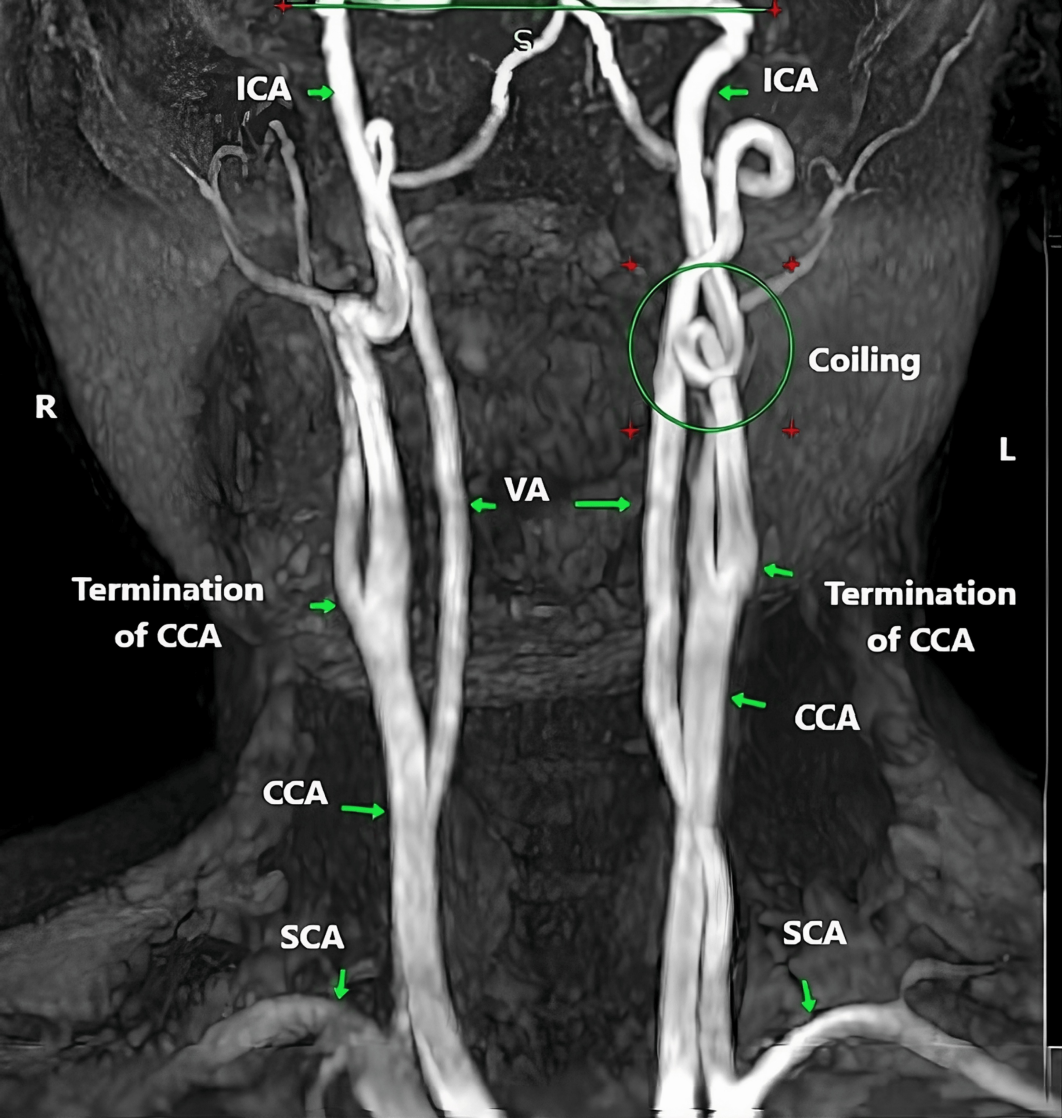
Left coiled type with a redundant looped configuration MRA of the neck vessels demonstrating dolichoarteriopathy of EICA. EICA - extracranial segment of the internal carotid artery; SCA - subclavian artery; CCA - common carotid artery; ECA - extracranial artery; VA - vertebral artery

A total of 197 EICA were evaluated bilaterally. Morphological variations were identified in 138 arteries, yielding an overall prevalence of 70.05%, while 59 arteries (29.94%) demonstrated a straight or normal course. Tortuosity was the most frequently observed variation, followed by kinking and coiling. The distribution of morphological patterns was comparable between the right and left sides, with no marked laterality predominance (Table 2, Figures 3-5).

**Table 2:** Presents the side-wise frequency distribution of morphological variations of the EICA This table presents the frequency and percentage distribution of morphological variations of the  EICA on the right and left sides, including straight (normal), tortuosity, kinking, and coiling. A total of 197 arteries were evaluated. Values are expressed as number and percentage (%). EICA - extracranial segment of the internal carotid artery

Morphological variation	Right EICA n (%)	Left EICA n (%)	Total n (%)
Straight	30 (30.6%)	29 (29.3%)	59 (29.94%)
Tortuosity	49 (50.0%)	47 (47.9%)	96 (48.73%)
Kinking	15 (15.3%)	17 (17.3%)	32 (16.24%)
Coiling	4 (4.1%)	6 (6.1%)	10 (5.07%)
Total	98 (100%)	99 (100%)	197 (100%)

Association of variations with age and sex

Age-wise analysis demonstrated a statistically significant association between EICA morphological variations and age (χ²=66.28, p<0.001). Patients aged >40 years showed a markedly higher prevalence of tortuosity and kinking compared with those aged ≤40 years. Odds ratio analysis confirmed significantly increased odds of tortuosity and kinking in individuals aged >40 years, while coiling showed no significant age association (Table 3).

**Table 3:** Presents the distribution of EICA morphological variations according to age and sex, along with odds ratios and confidence intervals Distribution of EICA morphological variations (normal, tortuosity, kinking, coiling) across age and sex in 197 arteries. Statistical analysis was performed using the Chi-squared or Fisher's exact test. Odds ratios (OR) with 95% confidence intervals (CI) are presented. A p-value <0.05 was considered significant. EICA - extracranial segment of the internal carotid artery

Variable	Normal n (%)	Tortuosity n (%)	Kinking n (%)	Coiling n (%)	χ²	OR (95% CI)	p-value
≤40 yrs	46 (77.96%)	19 (19.79%)	4 (12.5%)	7 (70%)	χ² = 66.28	Tortuosity: 14.34 (6.48-31.74), Kinking: 24.77 (7.35–83.49), Coiling: 1.52 (0.34-6.70)	<0.001
>40 yrs	13 (22.03%)	77 (80.20%)	28 (87.5%)	3 (30%)			
Male	42 (71.18%)	60 (62.5%)	23 (71.87%)	5 (50%)	χ² = 2.86	Tortuosity: 1.48 (0.74-2.98), Kinking: 0.97 (0.37–2.51), Coiling: 2.47 (0.63-9.64)	0.413
Female	17 (28.81%)	36 (37.5%)	9 (28.12%)	5 (50%)			

Sex-wise analysis did not reveal a significant association between EICA variations and gender (χ²=2.86, p=0.413). Although minor differences were observed in the distribution of individual variants between males and females, these were not statistically significant (Table 3).

Age differences in patients with and without variations

The mean age of patients with EICA morphological variations was significantly higher than that of patients without variations (51.57 ± 14.43 vs. 33.00 ± 13.08 years; t=8.84, p<0.001) (Table 4).

**Table 4:** Presents the Comparison of the mean age between patients with and without EICA morphological variations Comparison of mean age between patients with and without EICA morphological variations (n = 100). Values are presented as mean ± standard deviation (SD). Independent samples t-test was applied; p<0.05 was considered significant EICA - extracranial segment of the internal carotid artery

Group	Mean age ± SD	Test statistic	p-value
With variation	51.57 ± 14.43	t = 8.84, df ≈ 120.3	<0.001
Without variation	33.00 ± 13.08		

Severity of kinking (Metz classification)

**Figure 6:**
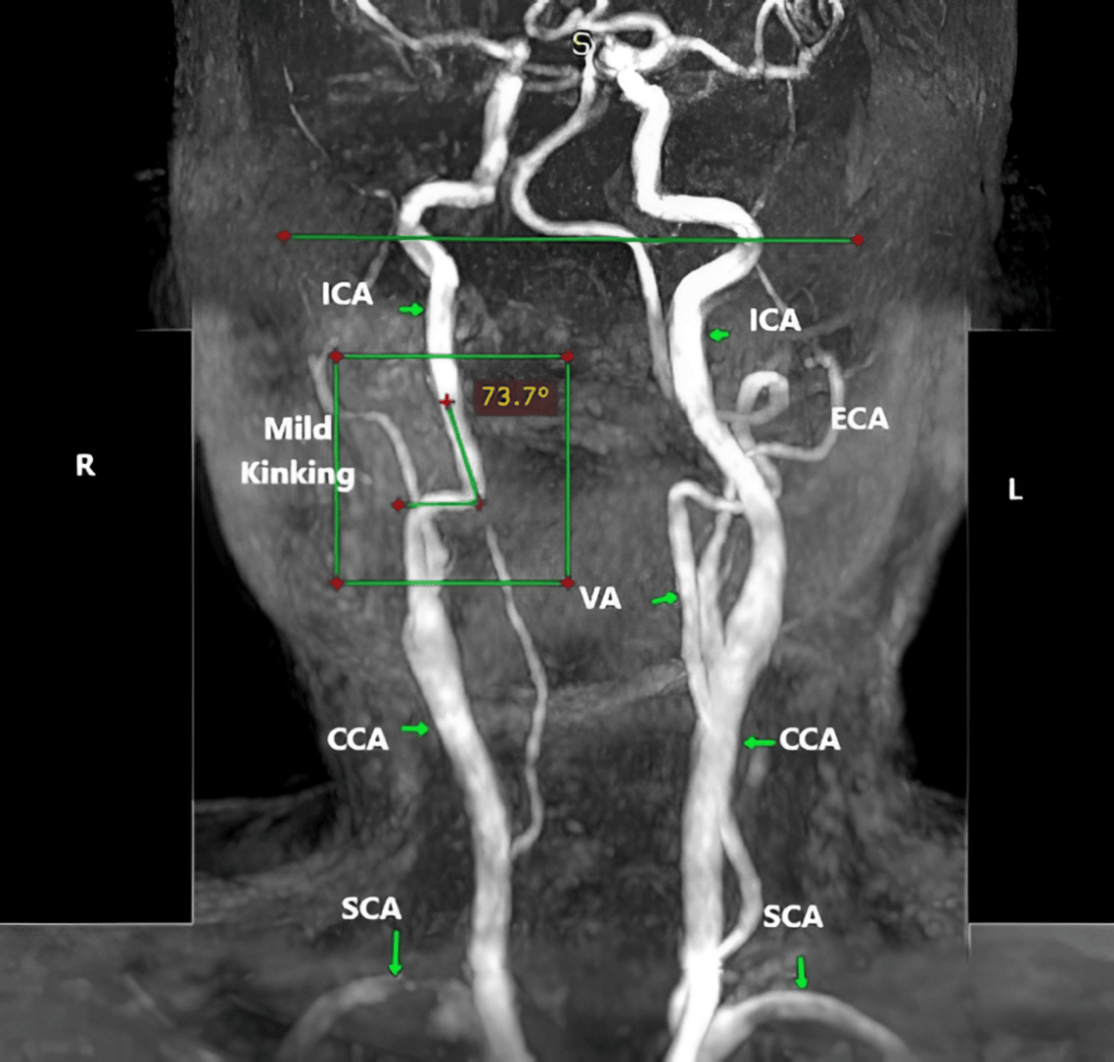
MRA depicting mild kinking of the left EICA characterized by an arterial angulation between 60° and 90° MRA of neck vessels showing severity of kinking. EICA - extracranial segment of the internal carotid artery; SCA - subclavian artery; ICA - internal carotid artery; CCA - common carotid artery; VA - vertebral artery

**Figure 7:**
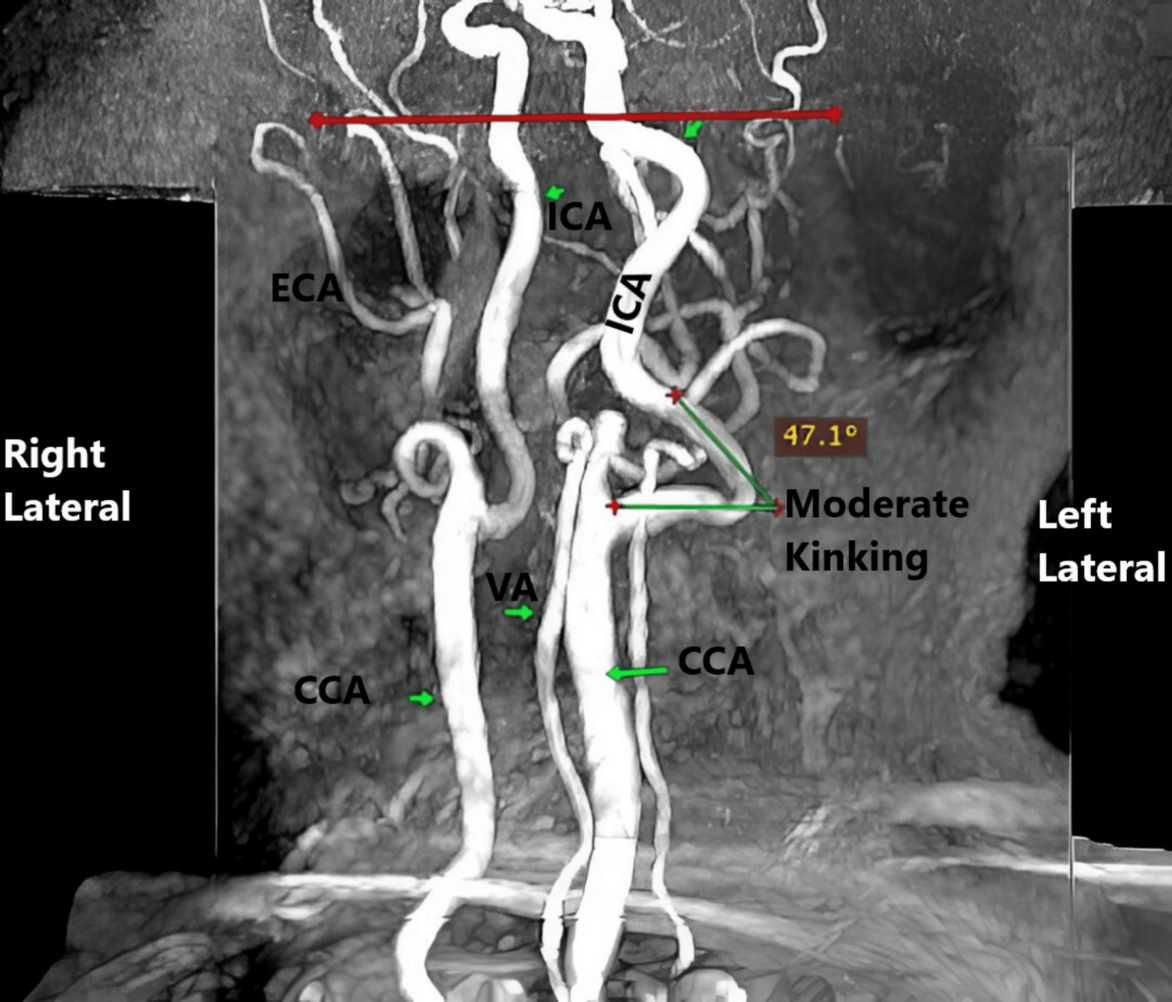
MRA depicting moderate kinking of the left EICA characterized by an arterial angulation between 30° and 60° EICA - extracranial segment of the internal carotid artery; ICA - internal carotid artery; CCA - common carotid artery; ECA - extracranial artery; VA - vertebral artery

**Figure 8:**
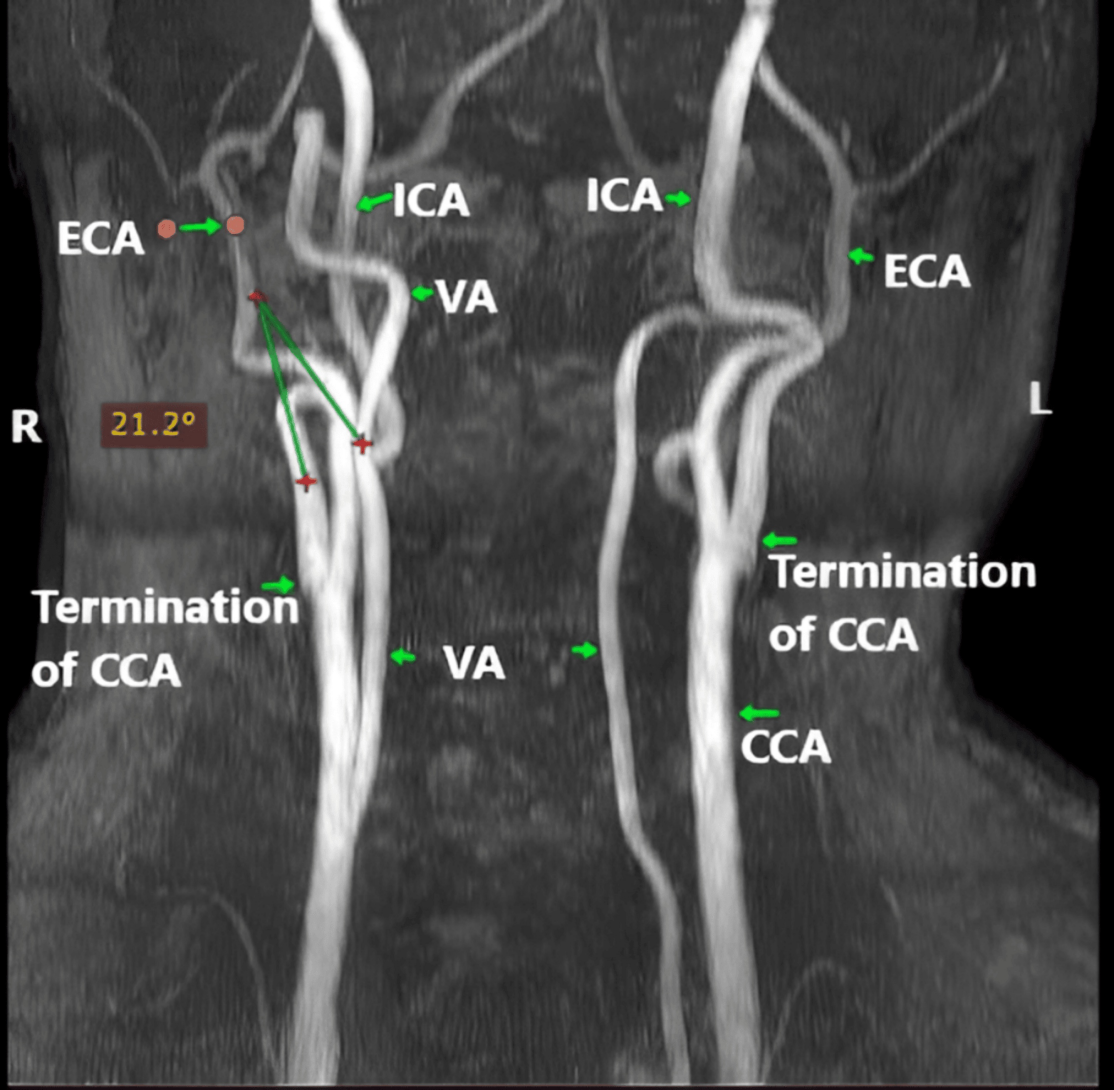
MRA depicting severe kinking of the left EICA characterized by an arterial angulation between 0° and 30° EICA - extracranial segment of the internal carotid artery; ICA - internal carotid artery; CCA - common carotid artery; ECA - extracranial artery; VA - vertebral artery

Kinking severity according to the Metz classification is presented in Table 5. Mild kinking was more common in patients aged ≤40 years compared to those >40 years, but it did not show a significant association with either age (χ²=2.81, p=0.246) or sex (χ²=0.59, p=0.745) (Figures 6-8).

**Table 5:** Presents the distribution of kinking severity across age groups and sex based on the Metz classification Distribution of kinking severity of the EICA according to the Metz classification across age groups (≤40 vs >40 years) and sex (male vs female) in 197 patients. Values are presented as number and percentage (%). Statistical comparisons were performed using the Chi-squared or Fisher's exact test. No significant association was observed between kinking severity and either age (χ²=2.81, p=0.246) or sex (χ²=0.59, p=0.745) EICA - extracranial segment of the internal carotid artery

Severity	≤40 yrs (n=4)	>40 yrs (n=28)	χ² / Fisher	p-value	Male (n=23)	Female (n=9)	χ² / Fisher	p-value
Mild	3 (75%)	16 (57.14%)	χ² = 2.81	0.246	15 (65.21%)	5 (55.55%)	χ² = 0.59	0.745
Moderate	0 (0%)	10 (35.71%)			7 (30.43%)	3 (33.33%)		
Severe	1 (25%)	2 (7.14%)			1 (4.34%)	1 (11.11%)		

## Discussion

In the present study, morphological variations of the EICA were identified in 70.05% of the 197 arteries evaluated, indicating that non-linear arterial configurations are more common than a straight course. Tortuosity was the predominant variant (48.73%), while kinking (16.24%) and coiling (5.07%) were less frequent. This pattern suggests that gradual arterial elongation and adaptive remodeling represent the dominant morphological response of the EICA, rather than focal angulation or looping. Although Desai and Toole described tortuosity, kinking, and coiling as common ICA variants, the marked predominance of tortuosity in the present cohort supports a stronger influence of progressive hemodynamic and structural adaptation [6].

Comparison with large CT angiography-based studies shows close agreement in overall prevalence, with reported EICA variation rates exceeding 70%. However, the relatively higher proportions of kinking and coiling observed in the present study may reflect differences in population characteristics or classification criteria rather than true epidemiological divergence. These variants demonstrate a clear tendency to increase with advancing age and show higher prevalence in females and hypertensive individuals, supporting the concept of vascular remodeling driven by chronic hemodynamic stress and systemic factors, as reported by Barfzadeh et al. [21]

Barfzadeh et al. (2024) reported a predominance of EICA tortuosity in older individuals and demonstrated associations with female sex and hypertension, supporting the concept that these variations are largely acquired rather than congenital. Similarly, Wang et al. observed a higher frequency of tortuosity in older populations, reinforcing age as a key determinant of carotid artery morphology [7]. These findings align with the present study, where advancing age emerged as the strongest factor associated with EICA variations. Collectively, these studies suggest that progressive arterial elongation, loss of elastic recoil, and cumulative hemodynamic stress contribute to age-related morphological remodeling of the carotid arteries rather than isolated focal deformities. Cantisano and Grubert further conceptualized such changes under the spectrum of dolichoarteriopathies, emphasizing that tortuosity and kinking tend to evolve and worsen over time as a consequence of vascular wall degeneration and altered flow dynamics [19]. Sun et al. similarly interpreted age-related increases in arterial tortuosity as part of physiological vascular aging, rather than inherently pathological changes, which may explain the high prevalence of asymptomatic cases observed in imaging-based studies [22].

With respect to kinking severity, Di Pino et al. demonstrated that mild forms are more frequently encountered, whereas moderate to severe kinking increases with advancing age, reflecting a continuum of arterial deformation rather than discrete entities [3]. In the present study, kinking severity did not differ significantly between males and females, a finding that is consistent with the observations of Togay-Işikay et al., who reported no meaningful sex-based differences in carotid artery kinking prevalence or severity [23].

Barfzadeh et al. identified female sex as a potential risk factor; however, they also emphasized the possibility of confounding by hypertension, suggesting that sex-related differences may not act independently. The proposed hormonal hypothesis-linking postmenopausal estrogen withdrawal to reduced arterial wall elasticity-offers a plausible mechanistic explanation, but current evidence remains inconclusive, underscoring the need for further targeted investigations to clarify this relationship [21].

Despite increasing research into coiling and kinking, many questions remain regarding their exact etiology, clinical role, and optimal management strategies. One proposed mechanism for neck artery kinking is that age-related reduction in intervertebral disc height and associated cervical spine degeneration may lead to relative arterial elongation and increased vessel curvature, as age-related spinal changes have been shown to decrease cervical flexibility and alter biomechanics [24].

From a clinical perspective, recognition of EICA morphological variations is crucial, as altered arterial geometry can significantly influence cerebral perfusion and local hemodynamics. Non-linear configurations such as tortuosity and kinking may predispose to turbulent blood flow, impaired distal perfusion, and increased technical difficulty during carotid endarterectomy, carotid artery stenting, head and neck surgery, and endovascular interventions [6,19]. Moderate to severe kinking, in particular, has been associated with compromised cerebral hemodynamics and a higher risk of ischemic events or peri-procedural complications, although a substantial proportion of cases remain clinically asymptomatic [3,23]. These observations underscore the complex relationship between anatomical variation and clinical manifestation. Consequently, routine assessment of EICA morphology using non-invasive imaging modalities, especially in older individuals, may play an important role in risk stratification and procedural planning. The age-related predominance of EICA variations observed in the present study further supports heightened attention to carotid geometry in elderly patients undergoing cervical or cerebrovascular interventions.

Limitations

This study has several limitations that should be acknowledged. First, its cross-sectional design precludes assessment of the temporal progression of EICA morphological changes, limiting causal inference regarding age-related vascular remodeling. The absence of longitudinal follow-up data prevents evaluation of whether observed variations progress in severity or contribute to future cerebrovascular events. Second, the study population consisted of patients undergoing MRA for non-specific neurological symptoms, which may introduce selection bias and limit generalizability to asymptomatic populations. Third, hemodynamic parameters and clinical outcome correlations were not assessed, restricting interpretation of the functional significance of the observed morphological variations. Future longitudinal studies incorporating flow analysis and clinical follow-up are warranted to better elucidate the prognostic implications of EICA morphological variants.

## Conclusions

Morphological variations of the extracranial internal carotid artery are common, with tortuosity being the most frequent pattern, followed by kinking and coiling. These variations show a strong association with increasing age, particularly in individuals over 40 years, whereas no significant association with sex was observed. From a clinical perspective, altered extracranial ICA geometry may modify cerebral blood flow dynamics and increase technical complexity and procedural risk during carotid endarterectomy, stenting, head and neck surgeries, and endovascular interventions. Awareness of these variations is therefore essential for radiologists, surgeons, and interventionalists during pre-procedural evaluation and surgical planning. Routine assessment of extracranial ICA morphology using non-invasive imaging modalities such as MR angiography may assist in risk stratification and procedural decision-making, especially in older patients. Future large-scale, multicenter studies incorporating longitudinal follow-up, hemodynamic assessment, and clinical outcome correlation are warranted to better clarify the vascular mechanisms underlying extracranial ICA dolichoarteriopathies and their broader clinical significance.

## References

[1] Bergin M, Bird P, Cowan I, Pearson JF (2010). Exploring the critical distance and position relationships between the Eustachian tube and the internal carotid artery. Otol Neurotol.

[2] Pfeiffer J, Becker C, Ridder GJ (2016). Aberrant extracranial internal carotid arteries: New insights, implications, and demand for a clinical grading system. Head Neck.

[3] Di Pino L, Franchina AG, Costa S (2021). Prevalence and morphological changes of carotid kinking and coiling in growth: an echo-color Doppler study of 2856 subjects between aged 0 to 96 years. Int J Cardiovasc Imaging.

[4] Gill JK, Sadiq M, Badar Z, Ezhapilli S (2017). Clinically significant anatomical variation of the retropharyngeal internal carotid arteries. Radiol Case Rep.

[5] WE J, FI WS (1965). Tortuosity, coiling, and kinking of the internal carotid artery. I. etiology and radiographic anatomy. Neurology.

[6] Desai B, Toole JF (1975). Kinks, coils, and carotids: a review. Stroke.

[7] Wang HF, Wang DM, Wang JJ (2017). Extracranial internal carotid artery tortuosity and body mass index. Front Neurol.

[8] Ekici F, Tekbas G, Onder H (2012). Course anomalies of extracranial internal carotid artery and their relationship with pharyngeal wall: an evaluation with multislice CT. Surg Radiol Anat.

[9] Beigelman R, Izaguirre AM, Robles M, Grana DR, Ambrosio G, Milei J (2010). Are kinking and coiling of carotid artery congenital or acquired?. Angiology.

[10] Cappabianca S, Somma F, Negro A, Rotondo M, Scuotto A, Rotondo A (2016). Extracranial internal carotid artery: anatomical variations in asymptomatic patients. Surg Radiol Anat.

[11] Milic DJ, Jovanovic MM, Zivic SS, Jankovic RJ (2007). Coiling of the left common carotid artery as a cause of transient ischemic attacks. J Vasc Surg.

[12] Najafi H, Javid H, Dye WS (1964). Kinked internal carotid artery. Clinical evaluation and surgical correction. Arch Surg.

[13] Pokrovskiĭ AV, Beloiartsev DF, Timina IE, Adyrkhaev ZA (2011). Clinical manifestations and diagnosis of pathological deformity of the internal carotid artery. Angiol Sosud Khir.

[14] Aleksic M, Schütz G, Gerth S, Mulch J (2004). Surgical approach to kinking and coiling of the internal carotid artery. J Cardiovasc Surg (Torino).

[15] Cartwright MS, Hickling WH, Roach ES (2006). Ischemic stroke in an adolescent with arterial tortuosity syndrome. Neurology.

[16] Chen YC, Wei XE, Lu J, Qiao RH, Shen XF, Li YH (2020). Correlation between internal carotid artery tortuosity and imaging of cerebral small vessel disease. Front Neurol.

[17] Zalewska-Adamiec M, Kuzma L, Bachorzewska-Gajewska H, Dobrzycki S (2021). Fractional flow reserve in the diagnosis of ischemic heart disease in a patient with coronary artery ectasia. Diagnostics (Basel).

[18] Ersoy H, Steigner ML, Coyner KB, Gerhard-Herman MD, Rybicki FJ, Bueno R, Nguyen LL (2012). Vascular thoracic outlet syndrome: protocol design and diagnostic value of contrast-enhanced 3D MR angiography and equilibrium phase imaging on 1.5- and 3-T MRI scanners. AJR Am J Roentgenol.

[19] Cantisano AL, Grubert RM (2023). Carotid artery kinking and tortuosity. Arq Bras Cardiol Imagem Cardiovasc.

[20] Metz H, Bannister RG, Murray-Leslie RM (1961). Kinking of the internal carotid artery. Lancet.

[21] Barfzadeh A, Saba M, Pourzand P, Jalalifar MR, Alizadeh SD, Mirkamali H, Rukerd MR (2024). Anatomical variations of the extracranial internal carotid artery: prevalence, risk factors, and imaging insights from CT-angiography. Surg Radiol Anat.

[22] Sun Z, Jiang D, Liu P (2022). Age-related tortuosity of carotid and vertebral arteries: quantitative evaluation with Mr angiography. Front Neurol.

[23] Togay-Işikay C, Kim J, Betterman K (2005). Carotid artery tortuosity, kinking, coiling: stroke risk factor, marker, or curiosity?. Acta Neurol Belg.

[24] Liebsch C, Greiner-Perth AK, Vogt M, Vieres V, Jonas R, Kienle A, Wilke HJ (2025). Intervertebral disc degeneration, age, and sex affect the range of motion of the cervical spine. Sci Rep.

